# Meat

**Published:** 1884-07

**Authors:** 


### Meat.

The value of meat as a food is due in a degree to its heat-producing properties, though in this respect it is surpassed by fatty and amyloid substances. It is as a tissue building material, and as an excitant of assimilative changes in the tissues, both with regard to itself and to non-nitrogenous foods, that it is most useful. It is stimulant as well as nutritive, and it therefore holds a deservedly high place in the daily dietary. Experiment has shown that three-quarters of a pound of lean meat fairly represents the quantity per diem which, taken with other less nitrogenous matter, suffices to maintain a person of average size and weight in a normal state of health. Some there are who largely exceed this standard, eating freely of meat at every meal, and living all the time quiet, sedentary lives. Such carnivorous feeders sooner or later pay a penalty by suffering attacks of gout or other disorders of indulgence. But it is equally important to note that many others, especially women, healthy in all points but for their innutrition, are apt to err as far on the other side. Thus one meets with people who consume about a pound of butcher's meat in a week, or not even that. This fact has been fully brought out by Dr. Graily Hewitt, in his address to the Obstetrical Section at the recent meeting of the British Medical Association. He has, likewise, with much probability, assigned this defect of diet as the chief cause of that general "weakness" which is so common among the antecedents of uterine displacement. The experience of many practitioners will confirm his observation. Different causes are at work to produce this kind of underfeeding—too rigid domestic economy, theoretical prejudices, the fastidious disinclination for food which comes of a languid indoor life without sufficient bodily exercise, tight lacing, perhaps, and many more. These difficulties are all more or less removable, unless, indeed, where absolute poverty forms the impediment. No effort should be spared to remove them. The advantages derived from a diet containing a fair amount of solid animal food could not be obtained from a purely vegetable or milk regimen without either unnecessarily burdening the digestive system with much surplus material, or, on the other hand, requiring such revolutionary changes as to quantity and quality of food and times of eating as would probably altogether prevent its general adoption, even were that desirable, into household management. In our opinion, such changes are not desirable, as being inadequate to secure their purpose.—*Lancet.*

